# Efficacy of Portable Air Cleaners and Masking for Reducing Indoor Exposure to Simulated Exhaled SARS-CoV-2 Aerosols — United States, 2021

**DOI:** 10.15585/mmwr.mm7027e1

**Published:** 2021-07-09

**Authors:** William G. Lindsley, Raymond C. Derk, Jayme P. Coyle, Stephen B. Martin, Kenneth R. Mead, Francoise M. Blachere, Donald H. Beezhold, John T. Brooks, Theresa Boots, John D. Noti

**Affiliations:** ^1^Health Effects Laboratory Division, National Institute for Occupational Safety and Health, CDC; ^2^Respiratory Health Division, National Institute for Occupational Safety and Health, CDC; ^3^Division of Field Studies and Engineering, National Institute for Occupational Safety and Health, CDC; ^4^CDC COVID-19 Response Team.

SARS-CoV-2, the virus that causes COVID-19, can be spread by exposure to droplets and aerosols of respiratory fluids that are released by infected persons when they cough, sing, talk, or exhale. To reduce indoor transmission of SARS-CoV-2 between persons, CDC recommends measures including physical distancing, universal masking (the use of face masks in public places by everyone who is not fully vaccinated), and increased room ventilation (*1*). Ventilation systems can be supplemented with portable high efficiency particulate air (HEPA) cleaners* to reduce the number of infectious particles in the air and provide enhanced protection from transmission between persons (*2*); two recent reports found that HEPA air cleaners in classrooms could reduce overall aerosol particle concentrations by ≥80% within 30 minutes (*3*,*4*). To investigate the effectiveness of portable HEPA air cleaners and universal masking at reducing exposure to exhaled aerosol particles, the investigation team used respiratory simulators to mimic a person with COVID-19 and other, uninfected persons in a conference room. The addition of two HEPA air cleaners that met the Environmental Protection Agency (EPA)–recommended clean air delivery rate (CADR) (*5*) reduced overall exposure to simulated exhaled aerosol particles by up to 65% without universal masking. Without the HEPA air cleaners, universal masking reduced the combined mean aerosol concentration by 72%. The combination of the two HEPA air cleaners and universal masking reduced overall exposure by up to 90%. The HEPA air cleaners were most effective when they were close to the aerosol source. These findings suggest that portable HEPA air cleaners can reduce exposure to SARS-CoV-2 aerosols in indoor environments, with greater reductions in exposure occurring when used in combination with universal masking.

A breathing aerosol source simulator was used to mimic a meeting participant exhaling infectious particles (source), and three breathing simulators were used to mimic a speaker and two participants exposed to these aerosol particles (receivers) (Figure 1). The methods used were similar to those used in previous studies of aerosol dispersion and transport in indoor spaces (*3*,*4*,*6*). The simulators were placed in a 584–ft^2^ (54–m^2^) conference room with a heating, ventilation, and air conditioning (HVAC) system that provided 0.1 m^3^ per second of air flow (202 ft^3^ per minute; two air changes per hour) with no air recirculation. Two HEPA air cleaners (Honeywell 50250-S, Kaz Inc.) were used, each rated to provide 250 ft^3^ per minute (0.12 m^3^ per second) of air filtration for a combined total of 5.2 air changes per hour. The two air cleaners were used in four different locations: 1) center of the room on the floor behind the source simulator; 2) left and right sides of the room on the floor; 3) left and right sides of the room and elevated 32 in (0.8 m); and 4) front and back of the room on the floor. Control experiments used no air cleaners.

**FIGURE 1:**
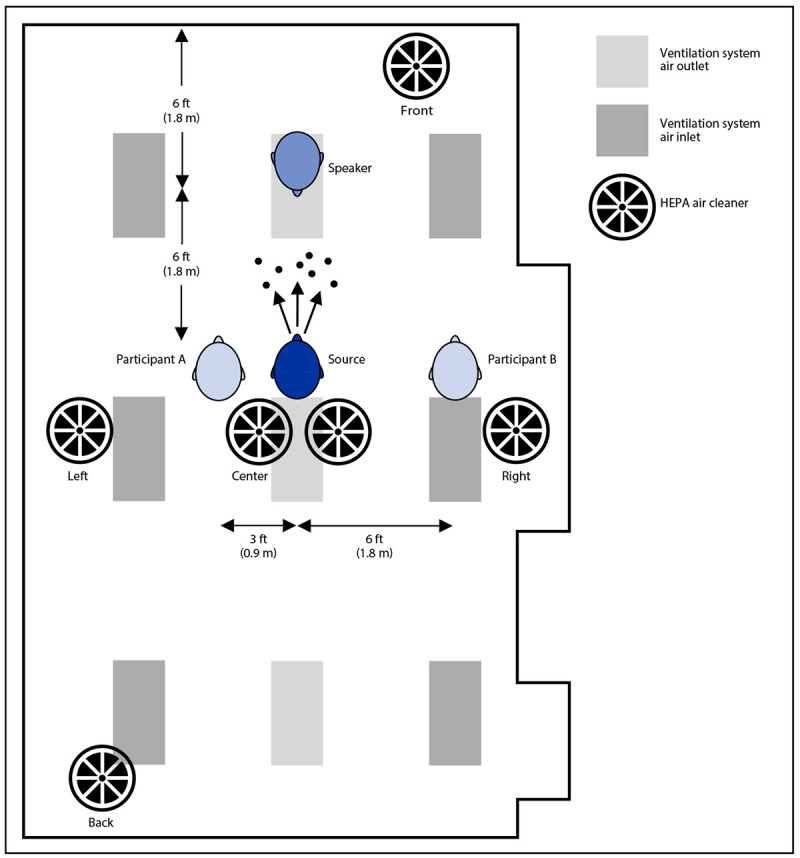
Representation of conference room* containing a breathing aerosol source simulator^†^ used to mimic a meeting participant exhaling infectious particles (source),^§^ and three breathing simulators used to mimic a speaker and two participants exposed to these aerosol particles (receivers) — United States, 2021^¶^ **Abbreviation:** HEPA = high efficiency particulate air. * The room is 21 ft (6.3 m) x 31 ft (9.3 m) x 10 ft (3 m). ^†^ The mouths of the participant source and participant receiver simulators were 40 in (1 m) above the floor, simulating persons sitting in a meeting or classroom. The mouth of the speaker receiver was 5 ft (1.5 m) above the floor, simulating a speaker standing in the front of the room. The air cleaners were placed either side-by-side in the center of the room on the floor, in the front and back of the room on the floor, on the left and right sides of the room on the floor, or on the left and right sides of the room and elevated 30 in (0.8 m). The room ventilation system air inlets and outlets were located in the ceiling as part of the light fixtures. ^§^ The source simulator breathed continuously at 15 liters per minute, and the aerosol generator was repeatedly cycled on for 20 seconds and off for 40 seconds to avoid exceeding the range of the aerosol instruments. ^¶^ Two participant breathing simulators (participant receivers) had a design based on the respiratory aerosol source simulator and breathed continuously at 15 liters per minute. The speaker breathing simulator (speaker receiver) was a commercial simulator that breathed at 28 liters per minute.

The source simulator (*6*) breathed continuously at 15 L/min. Two participant simulators (participant receivers) similar in design to the respiratory aerosol source simulator breathed continuously at 15 L/min. The speaker simulator (speaker receiver) was a commercial simulator (Warwick Technologies Ltd.) that breathed at 28 L/min. To mimic human heads, all simulators had headforms with elastomeric skin (source simulator headform, Hanson Robotics; receiver simulator headforms, Respirator Testing Head Form 1–Static, Crawley Creatures Ltd.). The face masks used on the headforms were three-ply cotton cloth face masks with ear loops (Defender, HanesBrands Inc.). Experiments were conducted either with all simulators unmasked or all simulators masked (universal masking).

The concentrations of 0.3 *μ*m to 3 *μ*m aerosol particles were measured at the mouth of each receiver using optical particle counters (Model 1.108, Grimm Technologies, Inc.) to determine the exposure of each receiver simulator to aerosol particles. When the simulators were masked, the particle counters collected aerosol samples from inside the masks (i.e., the particle counter measured the concentration of the aerosol being inhaled by the receiver simulator). For each optical particle counter, the total aerosol mass concentration was averaged over 60 minutes to determine the mean aerosol mass concentration (mean aerosol exposure) to which each receiver was exposed. Each experiment was repeated four times for a total of 20 tests. All data were analyzed using the Kruskal Wallis test to assess overall significance, followed by a Wilcoxon Rank Sum pairwise comparison with a Benjamini and Hochberg adjusted p-value for multiple comparisons. R software (version 3.6.0; R Foundation) was used to conduct all analyses.

The mean aerosol concentrations for the two participant receivers and the speaker receiver were generally similar during each experiment, indicating that the air in the room was well mixed over the 60-minute test period (Table). For all assessed scenarios, use of the HEPA air cleaners significantly reduced the aerosol exposures for the two participant receivers and speaker receiver (p = 0.001) (Figure 2). Without masks, the combined mean aerosol concentrations for the two participant receivers and speaker receiver were reduced by 49% with the air cleaners in the left and right elevated positions, 52% in the left and right floor positions, 55% in the front and back floor positions, and 65% in the center floor positions. The reductions with the air cleaners in the center floor position were higher than those with the air cleaners in the left/right or front/back positions (p<0.01). The aerosol concentrations when the air cleaners were in the left and right floor, left and right elevated, and front and back floor position results did not differ significantly from one another. Without the HEPA air cleaners, universal masking reduced the combined mean aerosol concentration by 72% (p<0.001). When both universal masking and the HEPA air cleaners were used, the combined mean concentrations for the two participant receivers and the speaker decreased by as much as 90% (p<0.001) (Table).

**TABLE:** Mean aerosol concentrations and standard deviations measured at the mouth of each simulator over 60 minutes at varying HEPA air cleaner locations, by masking status — United States, 2021

Simulator/Masking status	Mean aerosol concentrations at four HEPA air cleaner locations, % (SD)				
	No air cleaner	Left and right (elevated)	Left and right (floor)	Front and back (floor)	Center of room (floor)
**No masks**					
Participant A	99.8 (28.3)	62.1 (8.2)	61.0 (2.9)	40.7 (8.4)	33.3 (1.5)
Participant B	105.8 (7.7)	45.2 (1.7)	48.6 (1.9)	43.8 (1.2)	41.9 (1.4)
Speaker	94.4 (12.6)	44.7 (0.9)	33.4 (1.8)	50.0 (10.5)	30.8 (1.1)
Participants and speaker combined*	**100.0 (12.1)**	**50.7 (3.3)**	**47.7 (1.6)**	**44.8 (5.7)**	**35.3 (1.3)**
**Universal masking**					
Participant A	31.2 (2.4)	22.5 (9.2)	33.1 (4.0)	12.2 (3.6)	10.9 (2.3)
Participant B	32.7 (3.9)	13.7 (3.5)	11.4 (0.9)	13.4 (4.5)	12.8 (2.7)
Speaker	21.7 (2.2)	7.3 (0.7)	6.8 (0.7)	8.1 (2.7)	5.1 (1.2)
Participants and speaker combined*	**28.5 (2.8)**	**14.5 (4.3)**	**17.1 (1.7)**	**11.2 (3.6)**	**9.6 (2.1)**

**Abbreviations:** HEPA = high efficiency particulate air; SD = standard deviation.

* The values for participants and speaker combined represent the average of the results for the two participant receivers and the speaker receiver.

**FIGURE 2:**
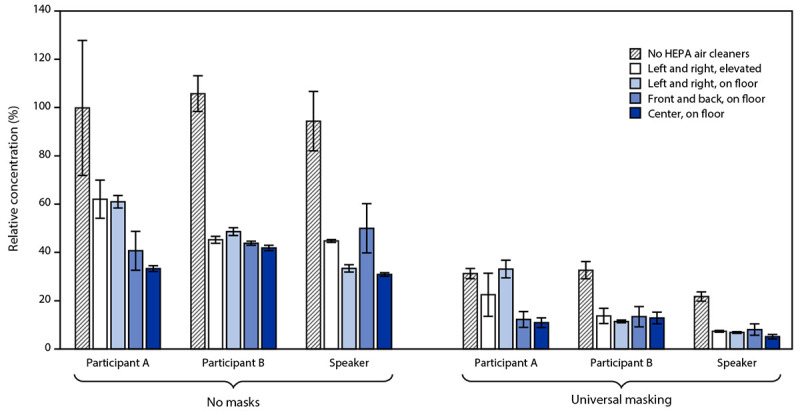
Concentrations* of aerosol particles at mouths of two participants and speaker relative to the combined average concentration measured for participants and speaker when high efficiency particulate air cleaners were not used and masks were not worn^†^ — United States, 2021 **Abbreviation:** HEPA = high efficiency particulate air. * The aerosol concentrations were measured at the mouths of two simulated participant receivers and simulated speaker receiver for 60 minutes while the simulated infected participant source exhaled aerosols into the room. ^†^ The legend indicates the locations of the HEPA air cleaners in the room. Each bar is the mean of four experiments. Error bars show the standard deviations.

## Discussion

In this study, the use of HEPA air cleaners in a conference room significantly reduced the exposure of nearby participants and a speaker to airborne particles produced by a simulated infected participant. The air cleaners were most effective when they were located in the center of the room close to the aerosol source. Moreover, the combination of HEPA air cleaners and universal masking was more effective than was either intervention alone. The use of masks without air cleaners reduced the aerosol exposure of the receivers by 72%, and the use of air cleaners without masks reduced the exposure by up to 65%. When used together, the HEPA air cleaners and masks reduced exposure to respiratory aerosols by up to 90%. These findings suggest that the use of portable HEPA air cleaners and universal masking can each reduce exposure to simulated SARS-CoV-2 aerosols in indoor environments, with larger reductions occurring when air cleaners and masking are used together.

Ventilation is a well-established method for reducing potential exposures to infectious aerosols (*7*). By removing airborne particles from a room, ventilation systems can reduce exposures that occur by inhalation of infectious aerosols, deposition on susceptible mucous membranes, or conveyance to mucous membranes by contaminated hands. However, in most nonclinical settings, ventilation systems are designed only with sufficient airflow to provide fresh air while maintaining comfortable temperature and humidity levels; these systems typically are not designed to have the much higher airflow rates that are needed to reduce disease transmission (*8*). During the ongoing pandemic, public health and professional organizations have provided guidance for increasing ventilation and air filtration to decrease the spread of SARS-CoV-2 (*2*,*9*,*10*). One recommended option, especially when existing HVAC systems might be insufficient, is adding portable HEPA air cleaners to rooms (*2*). The results of this study support the use of portable HEPA air cleaners to reduce exposure to airborne particles.

The findings in this report are subject to at least five limitations. First, the dispersion of aerosols in a room depends upon air currents, which are unique to each setting. In this study, the conference room air was well mixed, which helped transport aerosols to the air cleaners. In rooms with poor air mixing and potential stagnation zones, air cleaners might be less effective. Airflow patterns in real-world settings such as classrooms will vary among buildings and rooms, and rooms of different dimensions and with different ventilation rates will also have different airflow patterns. Second, the aerosol source manikin in this study was kept in one fixed location. In reality, potentially infectious occupants could be anywhere in the room and might move around the room occasionally. Third, this study only used one source manikin and three receiver manikins; additional sources and receivers could change the dynamics of aerosol dispersion within a room. Fourth, the study was limited to aerosol particles of 0.3 *μ*m to 3 *μ*m in size, which are small enough to remain airborne for an extended time but large enough to carry pathogens. However, particles outside this size range would behave differently. Finally, the study only assessed aerosol exposure; it did not directly examine disease transmission. Although the study provides useful information about the dynamics of respiratory aerosol particles and the effects of HEPA air cleaners and universal masking, many other factors are also important for disease transmission, including the amount of virus in the particles, how long the virus survives in air, and the vaccination status of the room occupants.

Portable HEPA air cleaners offer a simple means to increase the filtration of aerosol particles from a room without modifying the existing building ventilation system (*2*). The optimal location for HEPA air cleaners will depend upon the unique conditions in each room, but they are likely to be most effective when they are placed as close to the occupants as is practicable. Larger reductions in exposure occur when air cleaners are used in combination with universal masking. These findings support the utility of portable HEPA air cleaners and universal masking for reducing exposure to indoor aerosols containing SARS-CoV-2. Efforts to reduce SARS-CoV-2 aerosol exposure could help limit transmission of the virus and decrease incidences of COVID-19 illness and death.

**Summary**

**What is already known about this topic?**

Ventilation systems can be supplemented with portable high efficiency particulate air (HEPA) cleaners to reduce the number of airborne infectious particles.

**What is added by this report?**

A simulated infected meeting participant who was exhaling aerosols was placed in a room with two simulated uninfected participants and a simulated uninfected speaker. Using two HEPA air cleaners close to the aerosol source reduced the aerosol exposure of the uninfected participants and speaker by up to 65%. A combination of HEPA air cleaners and universal masking reduced exposure by up to 90%.

**What are the implications for public health practice?**

Portable HEPA air cleaners can reduce exposure to simulated SARS-CoV-2 aerosols in indoor environments, especially when combined with universal masking.

## Footnotes

* HEPA air cleaners consist of a filter capable of removing ≥99.97% of particles from the air and a fan or blower to draw air through the filter. HEPA air cleaners are commercially available, relatively inexpensive, and easy to use.

## References

[1] CDC. How COVID-19 spreads. Atlanta, GA: US Department of Human Services, CDC; 2021. Accessed June 22, 2021. https://www.cdc.gov/coronavirus/2019-ncov/prevent-getting-sick/how-covid-spreads.html

[2] CDC. Ventilation in buildings. Atlanta, GA: US Department of Human Services, CDC; 2021. Accessed May 10, 2021. https://www.cdc.gov/coronavirus/2019-ncov/community/ventilation.html

[3] Burgmann S, Janoske U. Transmission and reduction of aerosols in classrooms using air purifier systems. Phys Fluids (1994) 2021;33:033321. https://aip.scitation.org/doi/10.1063/5.0044046 10.1063/5.0044046 PMC8060972 33897240

[4] Curtius J, Granzin M, Schrod J. Testing mobile air purifiers in a school classroom: reducing the airborne transmission risk for SARS-CoV-2. Aerosol Sci Technol 2021;55:586–99. 10.1080/02786826.2021.1877257

[5] Environmental Protection Agency. Guide to air cleaners in the home: portable air cleaners furnace and HVAC filters Washington, DC: Environmental Protection Agency; 2018. https://www.epa.gov/sites/production/files/2018-07/documents/guide_to_air_cleaners_in_the_home_2nd_edition.pdf

[6] Lindsley WG, Beezhold DH, Coyle J, Efficacy of universal masking for source control and personal protection from simulated cough and exhaled aerosols in a room. J Occup Environ Hyg. Epub June 23, 2021. 10.1080/15459624.2021.1939879 PMC8355198 34161193

[7] Luongo JC, Fennelly KP, Keen JA, Zhai ZJ, Jones BW, Miller SL. Role of mechanical ventilation in the airborne transmission of infectious agents in buildings. Indoor Air 2016;26:666–78. 10.1111/ina.12267 26562748 PMC7165552

[8] Morawska L, Allen J, Bahnfleth W, A paradigm shift to combat indoor respiratory infection. Science 2021;372:689–91. 10.1126/science.abg2025 33986171

[9] Federation of European Heating, Ventilation and Air Conditioning Associations. REHVA COVID 19 guidance version 4.1. Brussels, Belgium: Federation of European Heating, Ventilation and Air Conditioning Associations; 2021. Accessed May 21, 2021. https://www.rehva.eu/rehva-covid-19-guidance-donation

[10] American Society of Heating, Refrigerating and Air-Conditioning Engineers. Coronavirus response resources from ASHRAE and others. Atlanta, GA: American Society of Heating, Refrigerating and Air-Conditioning Engineers; 2021. Accessed May 21, 2021. https://www.ashrae.org/technical-resources/resources

